# Supplementary material for: Site-Specific Dual-Labeling of a VHH with a Chelator and a Photosensitizer for Nuclear Imaging and Targeted Photodynamic Therapy of EGFR-Positive Tumors
Source: Cancers (Basel). 2021 Jan 23;13(3):428. doi: 10.3390/cancers13030428 (PMC7865570; doi:10.3390/cancers13030428)
Supplement: Supplementary file 1 [file cancers-13-00428-s001.pdf]

Supplementary material Site-Specific Dual-Labeling of a VHH with a Chelator and a Photosensitizer for Nuclear Imaging and Targeted Photodynamic Therapy of EGFR-Positive Tumors

Abbreviations 1
Materials and general procedures 1
Synthesis and analytical characterization of the bimodal probe DTPA-NH-Tz-S-IRDye700DX ... 6
Site-specific labeling of 7D12 with the bimodal probe 12
Spectral properties of compounds 16
Stability test by RP-HPLC in radiolabeling-like conditions......................................17

Abbreviations

BCN: Bicyclo[6.1.0]nonyne
CV: Column volume
DIPEA: *N,N*-Diisopropylethylamine
DMF: *N,N*-Dimethylformamide
DOL: Degree of labeling
DTPA: Diethylenetriaminepentaacetic acid
ESI: Electrospray ionization
FA: Formic acid
HATU: 1-[Bis(dimethylamino)methylene]-1H-1,2,3-triazolo[4,5-b]pyridinium 3-oxid hexafluorophosphate
HPLC: High performance liquid chromatography
HRMS: High resolution mass spectrometry
LRMS: Low resolution mass spectrometry
MALDI-TOF: Matrix-assisted laser desorption ionization - Time of flight
MES: 2-(*N*-morpholino)ethanesulfonic acid
NHS: *N*-hydroxysuccinimide
PB: Phosphate buffer
PBS: Phosphate buffered saline
PEG: Polyethylene glycol
RT: Room temperature
TCEP: Tris(2-carboxyethyl)phosphine
TEAB: Triethylammonium bicarbonate
TFA: Trifluoroacetic acid
THF: Tetrahydrofuran
TSTU: *N,N,N′,N′*-tetramethyl-*O*-(*N*-succinimidyl)uronium tetrafluoroborate
UV: Ultraviolet

Materials and general procedures

Chemicals. All chemicals were purchased from Sigma-Aldrich, Acros Organics, Alfa Aesar and TCI Europe and used without further purification. IRDye700DX-NHS was purchased from LICOR (USA). DTPA was kindly provided by CheMatech (France). Moisture sensitive reactions were performed under nitrogen or argon atmosphere. HPLC-gradient grade MeCN was obtained from Biosolve or Carlo Erba. LC-HRMS grade MeCN was obtained from Fisher Scientific. All aqueous buffers used in this work and aqueous mobile-phases for HPLC were prepared using water purified to 18.2 MΩ cm with a PURELAB Ultra system from ELGA.

Ion chromatography. The number of TFA counterions was quantified by ionic chromatography. Analyses were performed using an ICS 5000 system from Thermo Scientific equipped with a conductivity detector CD (Thermo Scientific)

and a conductivity suppressor ASRS-ultra II 4 mm (Thermo Scientific). TFA was separated using an Ion Pac AS11-HC analytical column (4 µm, 4 × 250 mm, Thermo Scientific) equipped with an Ion Pac AS11-HC guard column (4 µm, 4 × 250 mm, Thermo Scientific). Chromatographic analyses were recorded with Chromeleon software, 7.2. The elution of TFA was conducted with a 15 min isocratic elution with 90% eluent A (NaOH 30 mM) and 10% eluent B (H_2O) at a flow rate of 1.5 mL/min and a column temperature of 30 °C.

Semi-preparative HPLC. Purifications were performed on an UltiMate 3000 system Dionex (Thermo Scientific) equipped with an UV-visible detector, on one of the following columns: BetaBasic-18 column (Thermo Scientific; 5 µm, 150 Å, 150 × 30 mm) at 20 mL/min or Hypersil gold C18 column (Thermo Scientific, 100 Å, 250 × 10 mm) at 3.5 mL/min, with HPLC grade eluents. The fractions of interest were analyzed by HPLC-MS, pooled, concentrated under reduced pressure to remove organic solvents and freeze-dried.

HPLC-MS. The compounds were analyzed by HPLC-MS, performed on an UltiMate 3000 system Dionex (Thermo Scientific) equipped with a DAD detector and coupled to a low-resolution mass spectrometry detector MSQ Plus (Thermo Scientific) equipped with an ESI source. Separation was achieved using an RP Kinetex™ column (Phenomenex) (2.6 µm, 100 Å, 50 × 2.1 mm) with ultrapure water and HPLC-grade MeCN (A: H_2O 0.1% FA and B: MeCN 0.1% FA). Analyses were performed with the following gradient program: 5% to 100% of B in 5 min, 100% B for 1.5 min, 100% to 5% B in 0.1 min and 5% B for 1.9 min, at a flow rate of 0.5 mL/min. The purity of compounds was determined from the integration of RP-HPLC-MS chromatograms at 214 nm.

HPLC-MS chromatogram of H_2O injection (blank) at 214 nm:

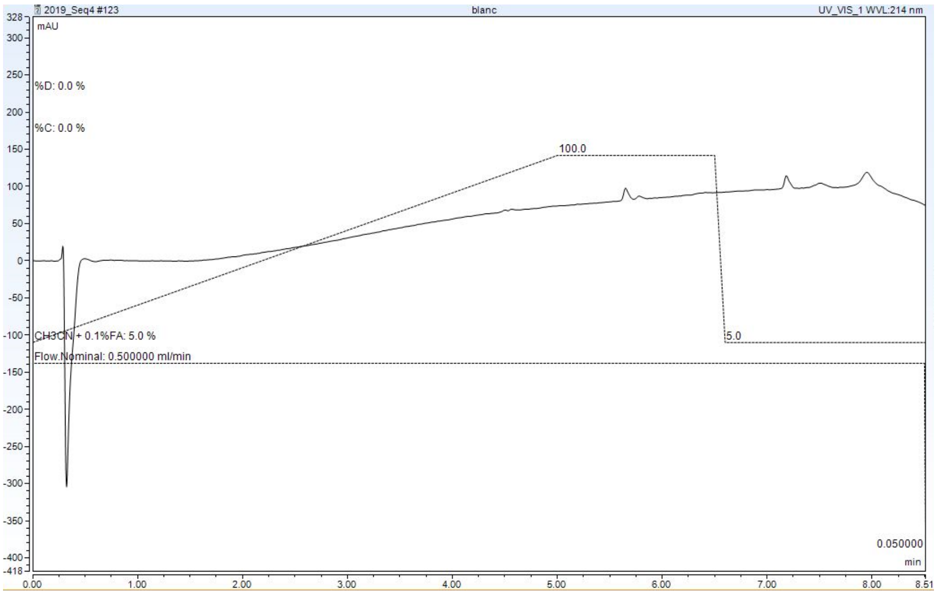

HRMS. Spectra were recorded on a mass spectrometer LTQ Orbitrap XL (Thermo Scientific) using an ESI source. Spectra were recorded in positive mode.

RP-HPLC-HRMS. The protein 7D12 and related conjugates were characterized by RP-HPLC-HRMS performed on a HPLC UltiMate 3000 system (Thermo Scientific) equipped with a UV-visible detector and coupled to a high-resolution mass spectrometer LTQ Orbitrap XL (Thermo Scientific) equipped with an ESI source. The separation was achieved on a Thermo Scientific™ MAbPac™ column (4 µm, 50 × 21 mm) with the following eluents: A: H_2O, 0.1% FA + 0.02% TFA and B: MeCN/H_2O (90 : 10, v/v), 0.1% FA + 0.02% TFA, and a linear gradient program: 5% to 100% of B in 15 min, 100% B for 3 min, 100% to 5% B in 0.1 min and 5% B for 6 min, at a flow rate of 0.3 mL/min, at 80 °C. Multicharged mass spectra were deconvoluted with Protein Deconvolution 2.0 software (Thermo Scientific).

MALDI-TOF. Analyses were recorded on a Bruker Daltonics Ultraflex II spectrometer equipped with a Smartbeam (Nd-YAG 355 nm) and used in the MALDI-TOF linear positive mode (10-25 kDa mass range). Samples were desalted

using C4 ZipTips, mixed with a freshly prepared 3,5-dimethoxy-4-hydrowycinnamic acid matrix solution and spotted on an MSP 365 MALDI plate.

^{1}H- and ^{13}C-NMR. Spectra were recorded on Bruker Avance Neo 500 MHz spectrometers under routine conditions at 298 K and were referenced to TMS or residual solvent signals. The following abbreviations and their combinations are used: s, singlet; d, doublet; t, triplet; q, quadruplet; m, multiplet. **Protein concentrations**. Concentrations of protein and protein conjugates were determined by measuring the absorbance on a Varian Cary 50 (double beam) spectrophotometer by using a TrayCell (Hellma, pathlength: 0.2 (factor 50) mm or 1.0 (factor 10) mm, chamber volume 0.7-4 µL), at 25 °C.**UV-visible spectra**. Spectra were obtained on an Agilent Cary 50 UV-Vis-Spectrophotometer by using a rectangular quartz cell (Hellma, 100-QS, 45 × 12.5 × 12.5 mm, pathlength: 10 mm, chamber volume: 3.5 mL), at 23 °C. Fluorescence spectroscopic studies were recorded on a JASCO FP8500 spectrofluorometer in a 10 mm path-length semi-micro quartz cuvette (Starna), at 25 °C.

Synthesis and analytical characterization of the bimodal probe DTPA-NH-Tz-S-IRDye700DX

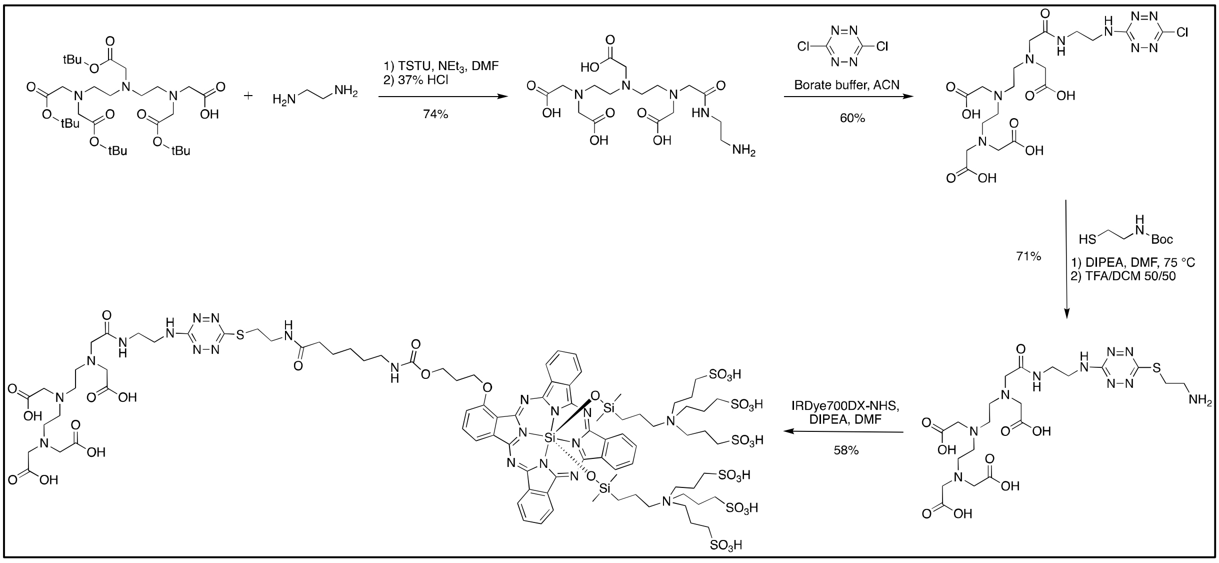

Figure S1. Synthesis of the bimodal probe DTPA-NH-Tz-S-IRDye700DX.

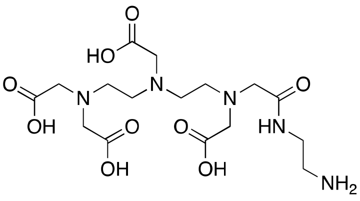

DTPA-NH$_2$. TSTU (121 mg, 0.402 mmol, 1.2 equiv.) was added to a solution of DTPA(tBu)$_4$ ester (207 mg, 0.335 mmol, 1 equiv.) in DMF (2 mL) with NEt$_3$ (140 µL, 1.005 mmol, 3 equiv.). The solution was stirred at room temperature for 1 h. Ethylenediamine (224 µL, 3.35 mmol, 10 equiv.) was then added to the activated ester and the reaction mixture was stirred for 2 h at room temperature to give a white suspension. This mixture was concentrated under vacuum and purified on semi-preparative HPLC on a BetaBasic-18 column (A: H_2O 0.1% TFA, B: MeCN 0.1% TFA; with the following gradient program: 5% of B for 10 min, 5% to 70% of B in 50 min, at a flow rate of 20 mL/min) to obtain a white solid. HCl 37% (6 mL) was added to the solid and the mixture was concentrated under vacuum. After lyophilization, the expected compound (brown solid) was obtained as a hydrochloride (3 HCl) salt (134.4 mg, 74%). The compound was analyzed with RP-HPLC-MS (Figure S2), ^{1}H- and ^{13}C-NMR and HRMS (Figure S3).

RP-HPLC-MS: t_r = injection peak, *m/z* calculated for $C_{16}H_{29}N_5O_9$ $[M+H]^+$ 436.2, found 436.3.

^{1}H-NMR (500 MHz, D_2O): δ = 3.1-3.25 (m, 6H), 3.45-3.6 (m, 6H), 3.68 (s, 2H), 4.14 (s, 4H), 4.19 (s, 4H) ppm; ^{13}C-NMR (126 MHz, D_2O): δ = 37.0 (CH_2), 38.8 (CH_2), 49.6 (CH_2), 49.9 (CH_2), 52.9 (CH_2), 53.9 (CH_2), 55.4 (CH_2), 56.2 (CH_2), 167.5 (C), 169.2 (C), 169.4 (C), 173.3 (C) ppm.

HRMS: *m/z* calculated for $C_{16}H_{29}N_5O_9$ $[M+H]^+$ 436.20447, found 436.20408.

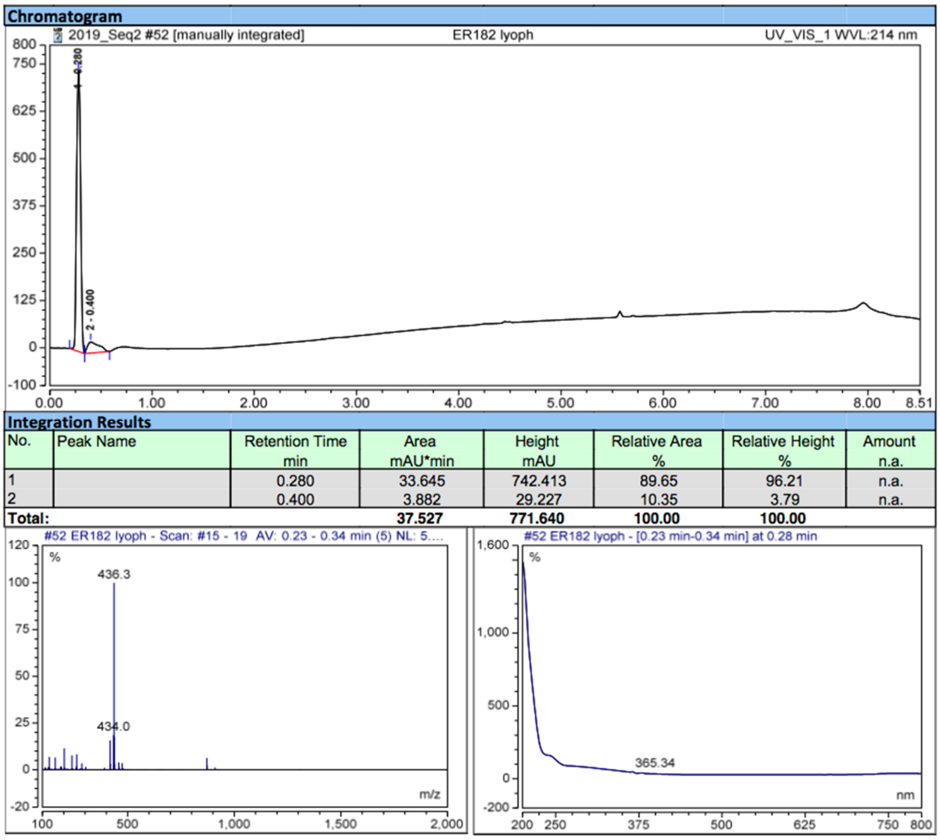

No.	Peak Name	Retention Time min	Area mAU*min	Height mAU	Relative Area %	Relative Height %	Amount n.a.
1		0.280	33.645	742.413	89.65	96.21	n.a.
2		0.400	3.882	29.227	10.35	3.79	n.a.
Total:			37.527	771.640	100.00	100.00	

Figure S2. RP-HPLC-MS analysis of DTPA-NH2. (Top) RP-HPLC chromatogram. (Bottom) ESI (+)-LRMS and UV-Vis absorbance spectrum.

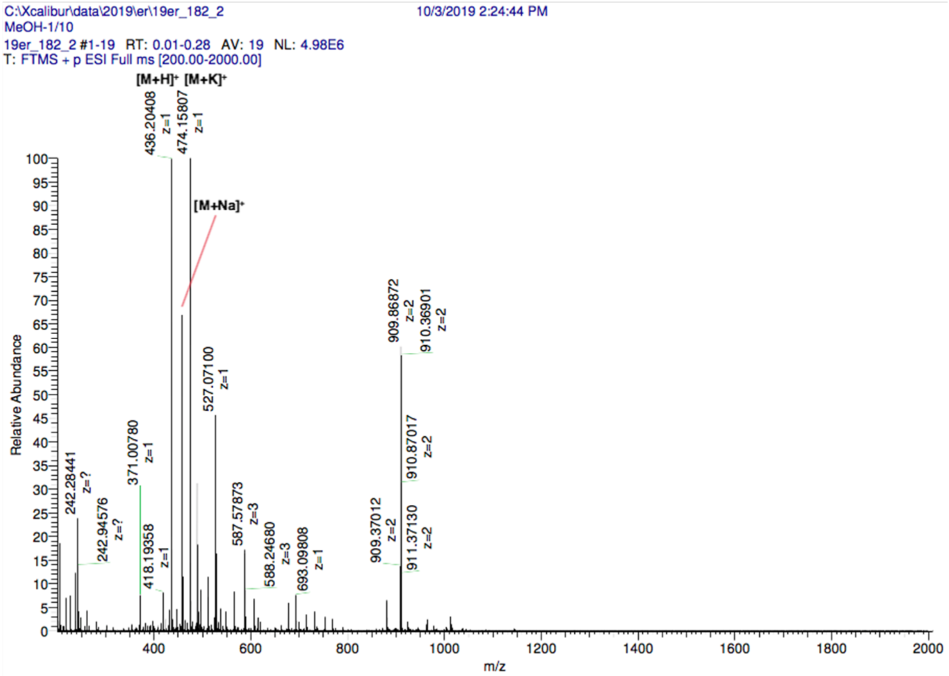

Figure S3. HRMS analysis of DTPA-NH2.

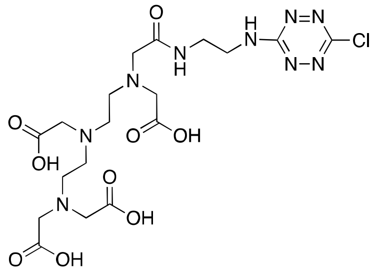

DTPA-NH-Tz-Cl. A solution of 3,6-dichloro-1,2,4,5-tetrazine (44.9 mg, 0.298 mmol, 1.2 equiv.) in acetonitrile (1 mL) was added to a solution of DTPA-NH_2 (134.4 mg, 0.248 mmol, 1 equiv.) in 0.1 M borate buffer pH 8.0 (4 mL). The resulting orange solution was stirred at room temperature for 1 h. The mixture was concentrated under vacuum and purified by semi-preparative HPLC on a BetaBasic-18 column (A: H_2O 0.1% TFA, B: MeCN 0.1% TFA; with the following gradient program: 2% of B for 10 min, 2% to 40% of B in 50 min, at a flow rate of 20 mL/min) to give an orange powder (98 mg, 60%). The compound was analyzed with RP-HPLC-MS (Figure S4), ^{1}H- and ^{13}C-NMR and HRMS (Figure S5).

Ionic chromatography: 1 TFA salt.

RP-HPLC-MS: tr = 0.45 min, m/z calculated for $C_{18}H_{28}ClN_9O_9$ [M+H]$^+$ 550.2, found 550.2.

^{1}H-NMR (500 MHz, D_2O): δ = 3.26 (t, J=5.8 Hz, 2H), 3.30 (t, J=6.0 Hz, 2H), 3.43 (t, J=5.8 Hz, 2H), 3.53 (t, J=6.1 Hz, 2H), 3.59 (t, J=5.4 Hz, 2H), 3.73 (t, J=5.5 Hz, 2H), 3.77 (s, 2H), 3.92 (s, 2H), 3.97 (s, 2H), 4.05 (s, 4H) ppm; ^{13}C-NMR (126 MHz, D_2O): δ = 38.4 (CH_2), 40.1 (CH_2), 50.3 (CH_2), 50.6 (CH_2), 52.2 (CH_2), 54.0 (CH_2), 55.8 (CH_2), 56.0 (CH_2), 56.4 (CH_2), 158.7 (C), 161.6 (C), 168.1 (C), 170.5 (C), 170.8 (C), 172.6 (C) ppm.

HRMS: m/z calculated for $C_{18}H_{28}ClN_9O_9$ [M+H]$^+$ 550.17779, found 550.17602.

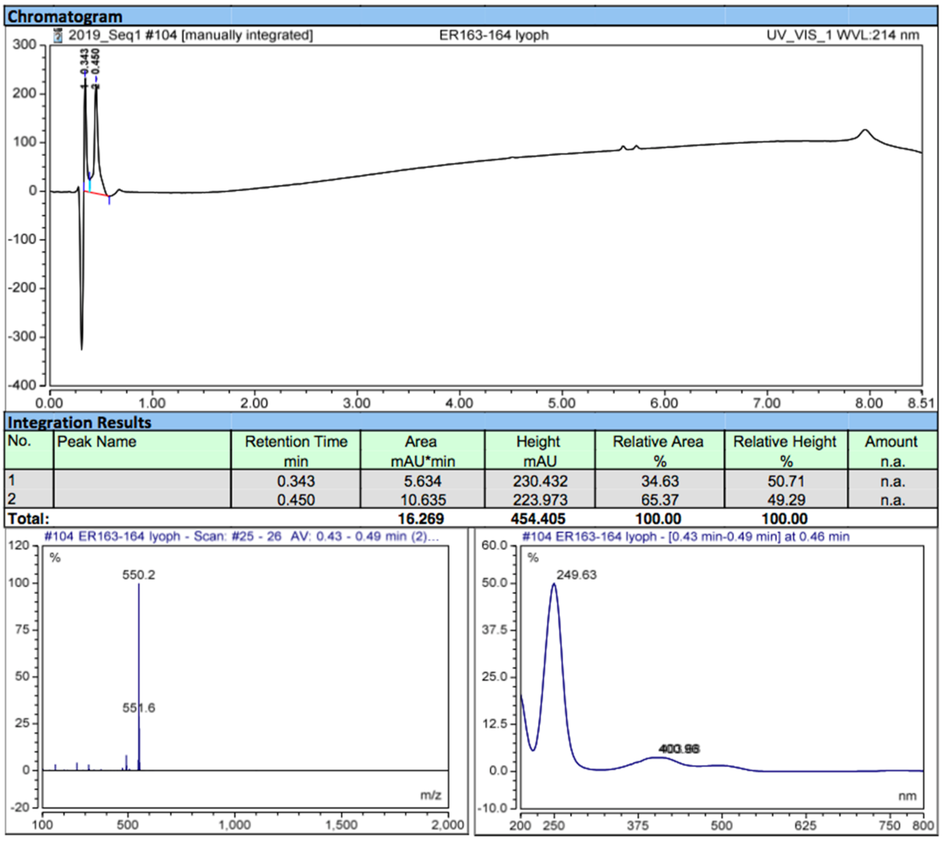

No.	Peak Name	Retention Time min	Area mAU*min	Height mAU	Relative Area %	Relative Height %	Amount n.a.
1		0.343	5.634	230.432	34.63	50.71	n.a.
2		0.450	10.635	223.973	65.37	49.29	n.a.
Total:			16.269	454.405	100.00	100.00	

Figure S4. RP-HPLC-MS analysis of DTPA-NH-Tz-Cl (Top) RP-HPLC chromatogram. (Bottom) ESI (+)-LRMS and UV-Vis absorbance spectrum.

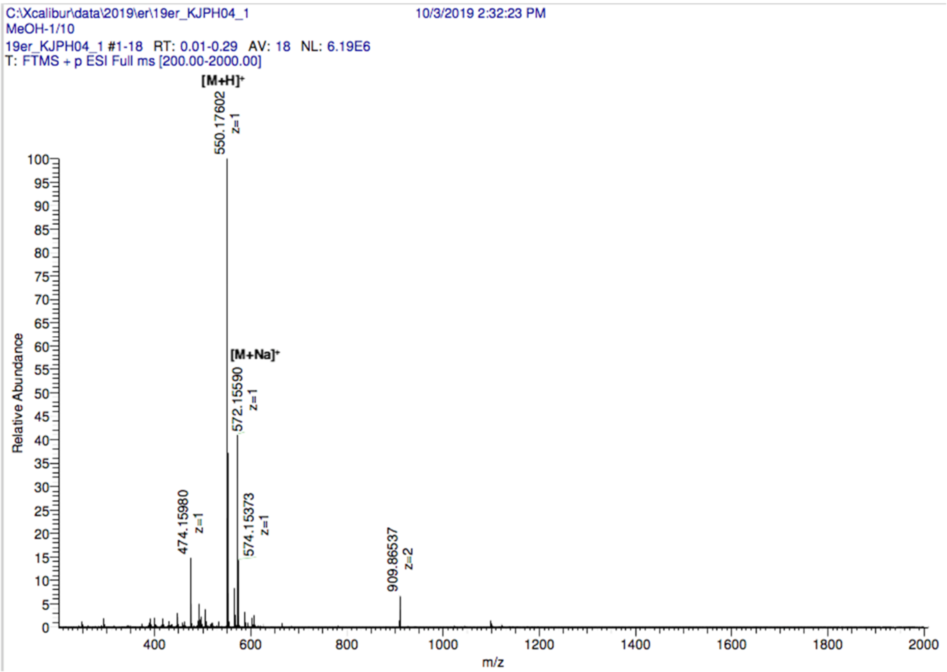

Figure S5. HRMS analysis of DTPA-NH-Tz-Cl.

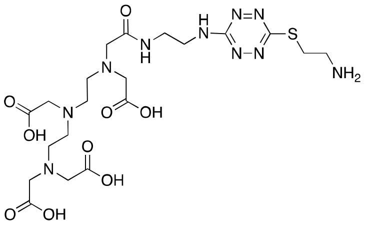

DTPA-NH-Tz-NH₂. Tert-butyl (2-mercaptoethyl)carbamate (14.9 mg, 84.1 µmol, 1.2 equiv.) in DMF (2 mL) was added to a solution of DTPA-NH-Tz-Cl (50 mg, 75.3 µmol, 1 equiv.) in DMF (2 mL) and DIPEA (104.5 µL, 602 µmol, 8 equiv.). The resulting orange solution was stirred at 75 °C. After 2 h, the solvent was removed under vacuum. CH_2Cl_2 (2 mL) and TFA (2 mL) were added and the resulting solution was stirred at room temperature for 2 h. After concentration under vacuum the mixture was purified by semi-preparative HPLC on a BetaBasic-18 column (A: H_2O 0.1% TFA, B: MeCN 0.1% TFA; with the following gradient program: 2% of B for 10 min, 2% to 40% of B in 50 min, at a flow rate of 20 mL/min) to afford an orange powder (43.8 mg, 71%). The compound was analyzed with RP-HPLC-MS (Figure S6), ^{1}H- and ^{13}C-NMR and HRMS (Figure S7).

Ionic chromatography: 2 TFA salt.

RP-HPLC-MS: t_r = 0.35 min, *m*/*z* calculated for $C_{20}H_{34}N_{10}O_9S$ [M+H]$^+$ 591.2, found 591.4.

^{1}H-NMR (500 MHz, D_2O): δ = 3.27 (m, 4H), 3.43 (m, 4H), 3.55 (m, 6H), 3.70 (m, 2H), 3.76 (s, 2H), 3.91 (s, 2H), 3.98 (s, 2H), 4.05 (s, 4H) ppm; ^{13}C-NMR (126 MHz, D_2O): δ = 27.8 (CH_2), 38.3 (CH_2), 38.8 (CH_2), 39.9 (CH_2), 50.3 (CH_2), 50.6 (CH_2), 52.2 (CH_2), 54.0 (CH_2), 55.8 (CH_2), 56.0 (CH_2), 56.4 (CH_2), 161.0 (C), 163.9 (C), 168.1 (C), 170.4 (C), 170.7 (C), 172.6 (C), ppm.

HRMS: *m*/*z* calculated for $C_{20}H_{34}N_{10}O_9S$ [M+H]$^+$ 591.23103, found 591.22921.

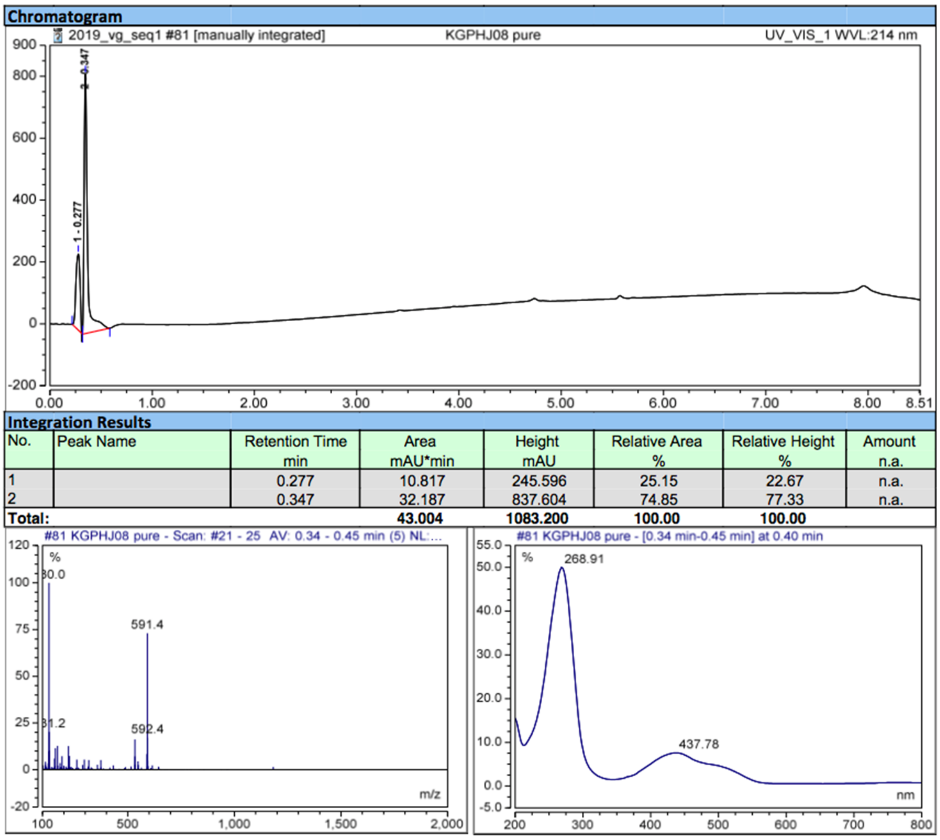

No.	Peak Name	Retention Time min	Area mAU*min	Height mAU	Relative Area %	Relative Height %	Amount n.a.
1		0.277	10.817	245.596	25.15	22.67	n.a.
2		0.347	32.187	837.604	74.85	77.33	n.a.
Total:			43.004	1083.200	100.00	100.00	

Figure S6. RP-HPLC-MS analysis of DTPA-NH-Tz-NH_2. (Top) RP-HPLC chromatogram. (Bottom) ESI (+)-LRMS and UV-Vis absorbance spectrum.

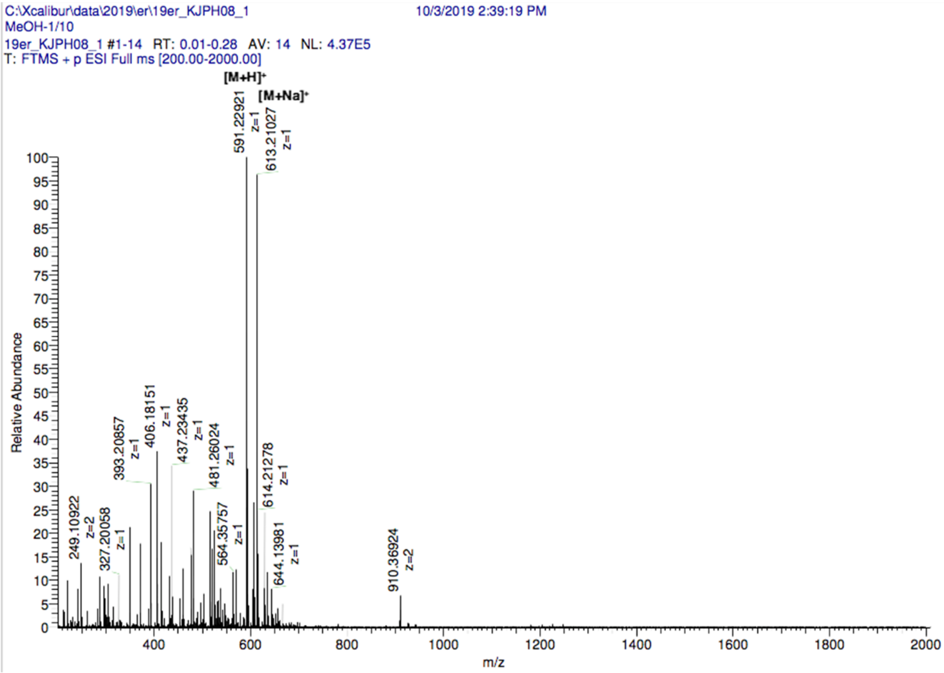

Figure S7. HRMS analysis of DTPA-NH-Tz-NH_2.

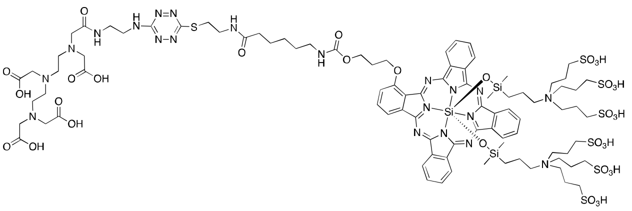

DTPA-NH-Tz-S-IRDye700DX. IRDye700DX (6.3 mg, 3.22 µmol, 1 equiv.) in DMF (1 mL) with DIPEA (8 µL, 45.9 µmol, 14 equiv.) was added to a suspension of DTPA-NH-Tz-NH_2 (3.43 mg, 4.19 µmol, 1.3 equiv.) in DMF (1 mL) with DIPEA (8 µL, 45.9 µmol, 14 equiv.). The green solution was stirred at RT overnight. After concentration under vacuum, the mixture was purified by semi-preparative HPLC on an Hypersil column (A: TEAB 50 mM, B: MeCN with the following gradient program: 20% of B for 5 min, 20% to 60% of B in 60 min, at a flow rate of 3.5 mL/min) to give a blue/green powder (4.4 mg, 58%). The compound was analyzed with RP-HPLC-MS (Figure S8).

RP-HPLC-MS: t_r = 3.6 min., *m/z* calculated for $C_{90}H_{129}N_{21}O_{33}S_7Si_3$ $[M+2H]^{2+}$ 1170.8, found 1171.4, *m/z* calculated for $[M+3H]^{3+}$ 780.9, found 779.2.

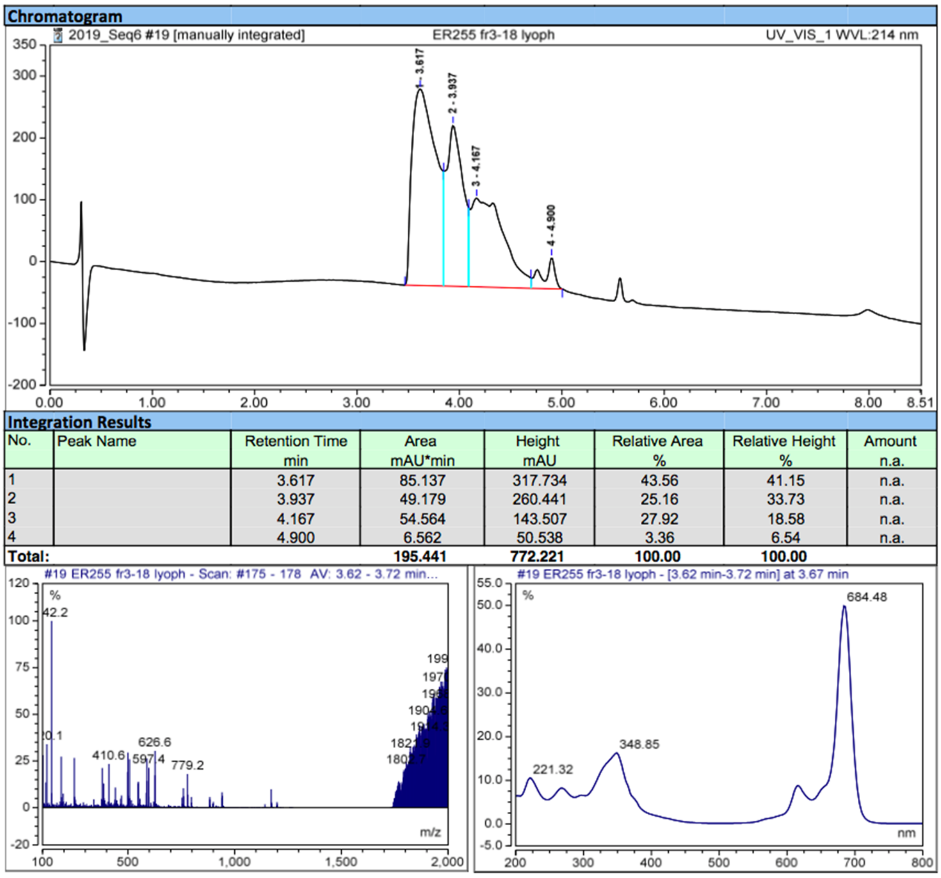

No.	Peak Name	Retention Time min	Area mAU*min	Height mAU	Relative Area %	Relative Height %	Amount n.a.
1		3.617	85.137	317.734	43.56	41.15	n.a.
2		3.937	49.179	260.441	25.16	33.73	n.a.
3		4.167	54.564	143.507	27.92	18.58	n.a.
4		4.900	6.562	50.538	3.36	6.54	n.a.
Total:			195.441	772.221	100.00	100.00	

Figure S8. RP-HPLC-MS analysis of compound DTPA-NH-Tz-S-IRDye700DX (Top) RP-HPLC chromatogram. (Bottom) ESI (+)-LRMS and UV-Vis absorbance spectrum.

Site-specific labeling of 7D12 with the bimodal probe

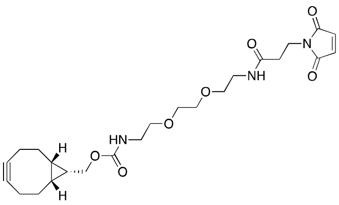

BCN-PEG$_2$-maleimide. A solution of maleimide-NHS (13.5 mg, 50.1 µmol, 1.1 equiv.) in DMF (0.5 mL) was added to a solution of BCN-PEG$_2$-NH_2 (15 mg, 46 µmol, 1 equiv.) in DMF (0.5 mL) and DIPEA (16.1 µL, 92 µmol, 2 equiv.). The resulting yellowish solution was stirred at RT. After 1 h, the reaction mixture was concentrated under vacuum and purified by semi-preparative HPLC on a BetaBasic-18 column (A: H_2O 0.1% FA, B: MeCN 0.1% FA; with the following gradient program: 15% of B for 5 min, 15% to 65% of B in 50 min, at a flow rate of 20 mL/min) to give a colorless oil (9.1 mg, 41%, purity: 87%). The compound was analyzed with RP-HPLC-MS (Figure S9), ^{1}H- and ^{13}C-NMR and HRMS (Figure S10).

Note: this compound proved to be unstable, giving rise to unidentified products over time, even when stored at -80 °C. It was aliquoted as a 20 mM solution in DMSO and kept at -20 °C.

RP-HPLC-MS: t_r = 4 min, *m/z* calculated for $C_{24}H_{33}N_3O_7$ $[M+H]^+$ 476.2, found 476.3.

^{1}H-NMR (500 MHz, $CDCl_3$): δ = 0.8-1.0 (m, 3H), 1.25 (m, 4H), 2.1-2.3 (m, 4H), 2.53 (t, J=7.2 Hz, 2H), 3.3-3.7 (m, 10H), 3.84 (t, J=7.2 Hz, 2H), 4.1 (m, 2H), 5.22 (br, 1H), 6.12 (br, 1H), 6.70 (d, 2H) ppm.

HRMS: *m/z* calculated for $C_{24}H_{33}N_3O_7$ $[M+H]^+$ 476.23185, found 498.22166.

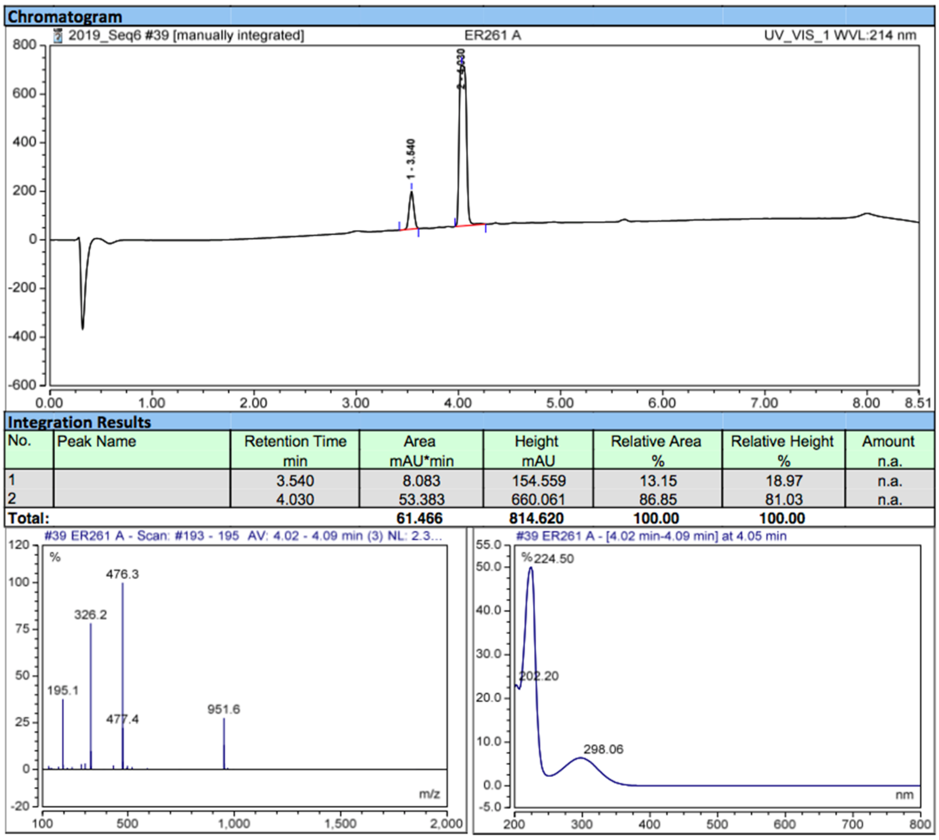

Figure S9. RP-HPLC-MS analysis of BCN-PEG2-maleimide. (Top) RP-HPLC chromatogram. Note: the impurity at tr = 3.54 min corresponds to the hydrolyzed or hydrated product (mass m/z= 494). (Bottom) ESI (+)-LRMS and UV-Vis absorbance spectrum.

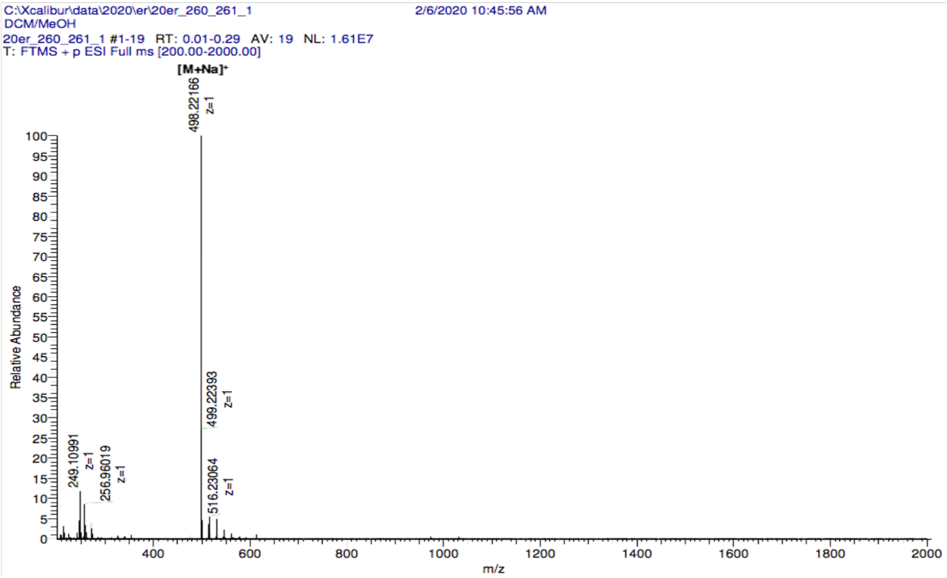

Figure S10. HRMS analysis of BCN-PEG2-maleimide.

7D12-BCN.

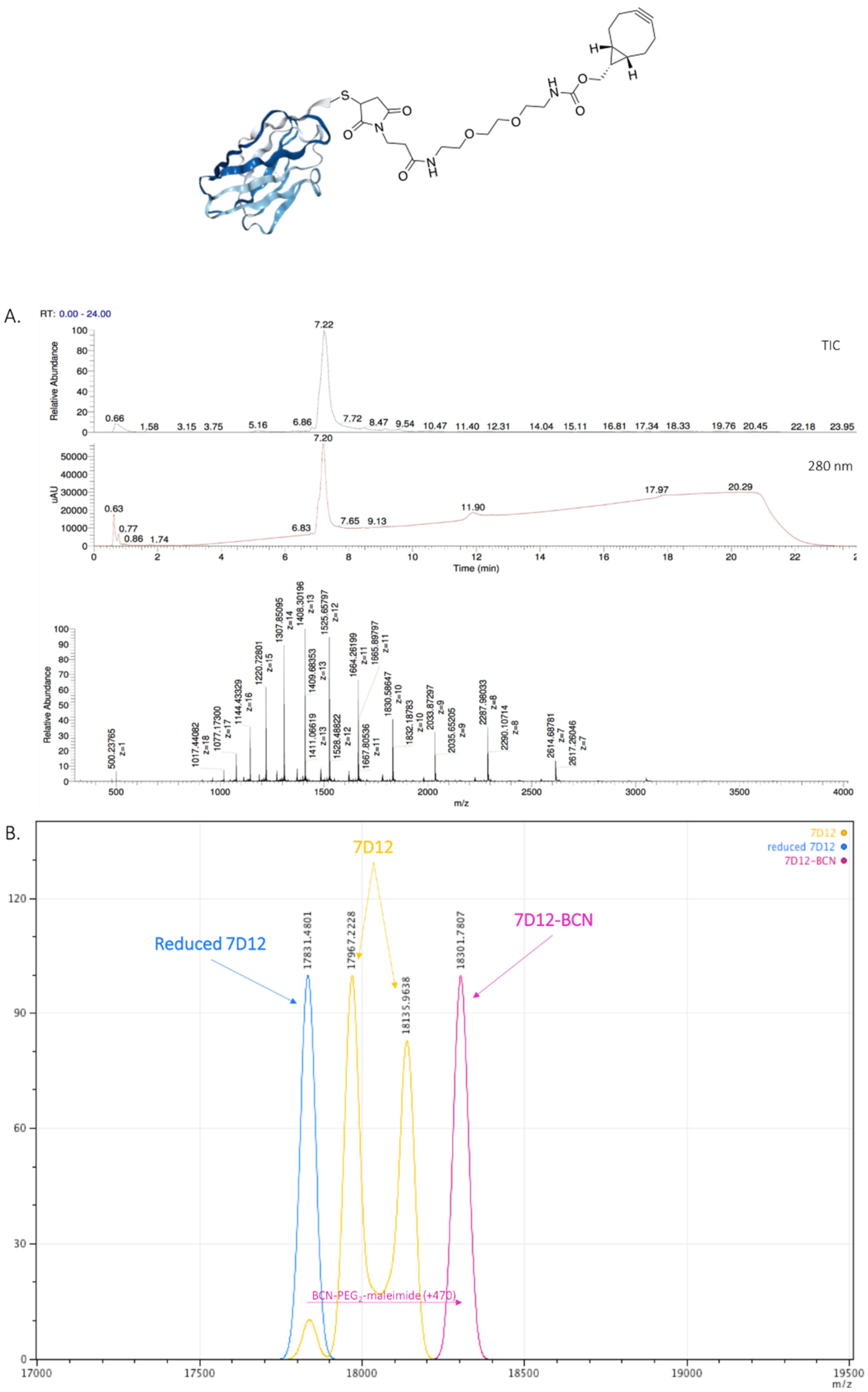

Figure S11. RP-HPLC-MS (Orbirap) analysis of compound 7D12-BCN. A. (Top) RP-HPLC chromatograms (TIC and 280 nm). (Bottom) Multicharged ESI-MS spectrum. B. Deconvoluted MS spectrum of native 7D12, reduced 7D12 and 7D12-BCN.

DTPA-IRDye700DX-7D12

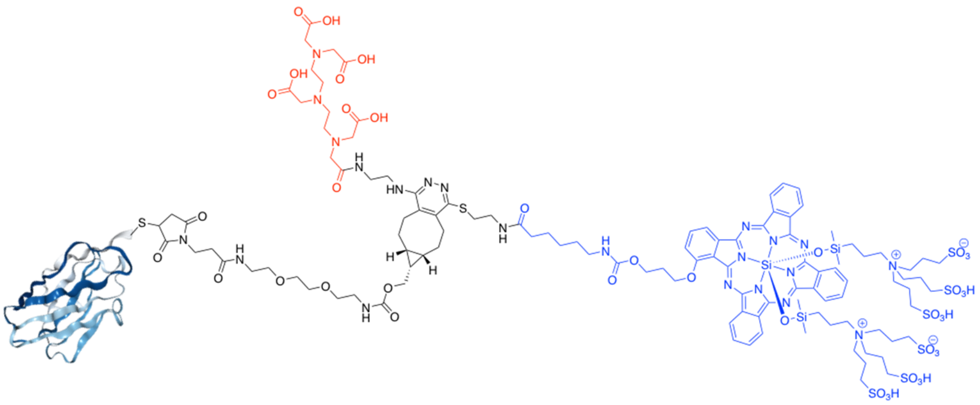

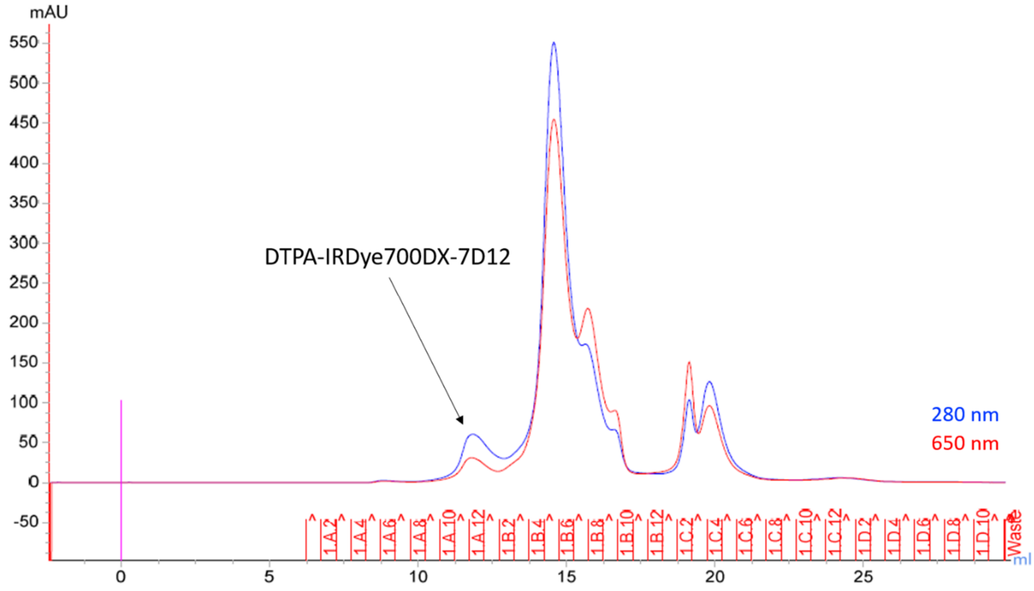

Figure S12. FPLC purification chromatogram of the compound DTPA-IRDye700DX-7D12.

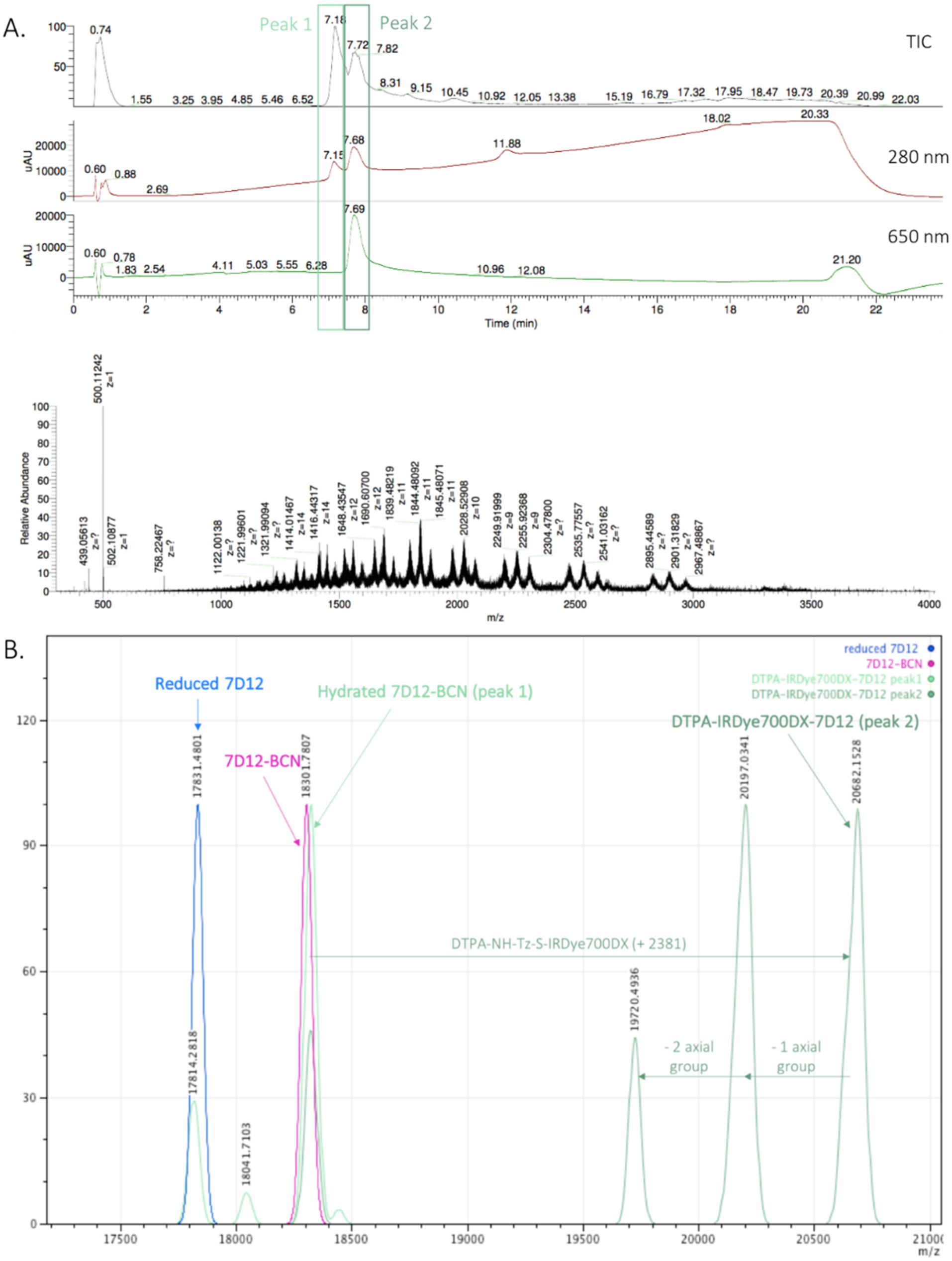

Figure S13. RP-HPLC-MS (Orbirap) analysis of compound DTPA-IRDye700DX-7D12. A. (Top) RP-HPLC chromatograms (TIC, 280 nm and 650 nm). (Bottom) Multicharged ESI-MS spectrum. B. Deconvoluted MS spectrum of reduced 7D12, 7D12-BCN and DTPA-IRDye700DX-7D12.

Spectral properties of compounds

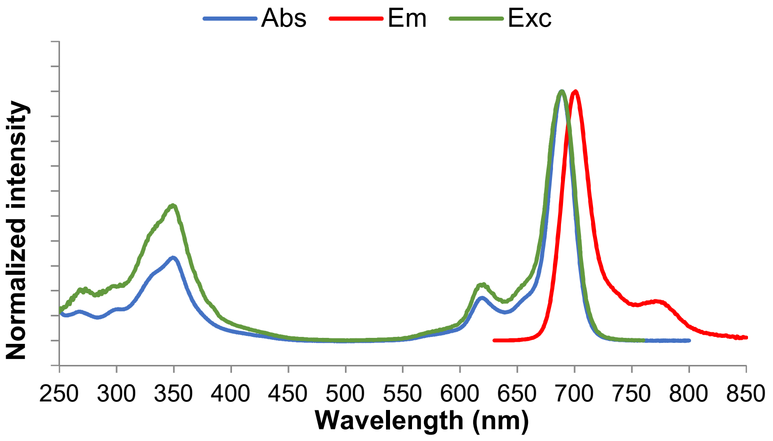

Figure S14. Normalized photophysical spectra (absorbance, excitation and emission) of compound IRDye700DX. $\lambda_{Ex, max}$ = 689 nm, $\lambda_{Em, max}$ = 701 nm.

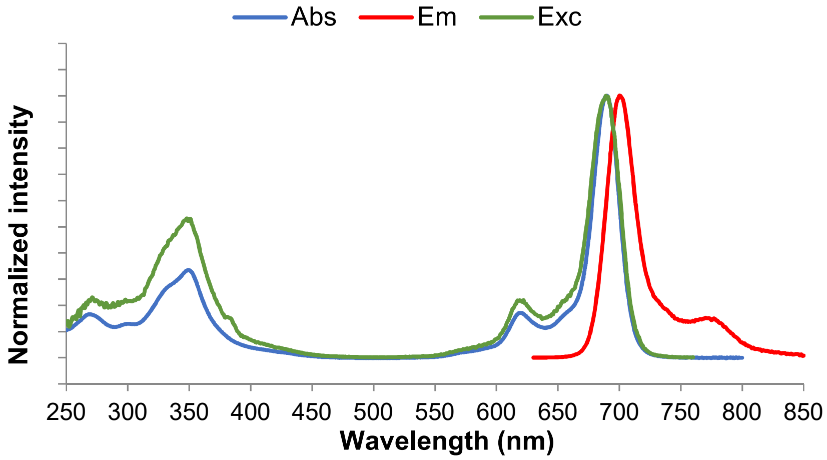

Figure S15. Normalized photophysical spectra (absorbance, excitation and emission) of bimodal probe DTPA-NH-Tz-S-IRDye700DX. $\lambda_{Ex, max}$ = 689 nm, $\lambda_{Em, max}$ = 700 nm

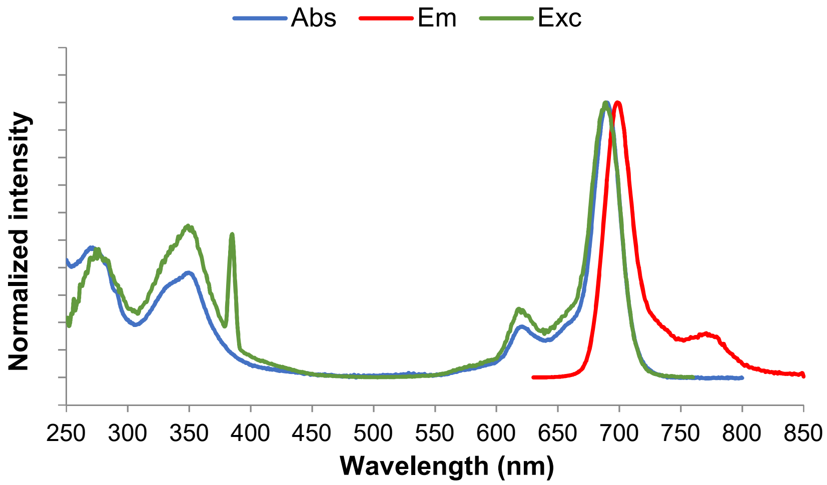

Figure S16. Normalized photophysical spectra (absorbance, excitation and emission) of bimodal conjugate DTPA-IRDYe700DX-7D12. $\lambda_{Ex, max}$ = 689 nm, $\lambda_{Em, max}$ = 698 nm

Stability test by RP-HPLC in radiolabeling-like conditions

8.3 µL of 0.05 M HCl and 16.6 µL of 0.5 M MES were added to a solution of DTPA-IRDye700DX-7D12 in PBS (1.04 mg/mL, 20 µg). The resulting solution was stirred in a thermomixer (900 rpm, 25 °C). After 0 min, 1 h, 2 h, 4 h a sample (15 µL) of solution was collected and analyzed by RP-HPLC-MS (Orbitrap) at a wavelength of 280 nm and 650 nm (Figure S17). The separation was achieved on a Thermo Scientific™ MAbPac™ column (4 µm, 50 × 21 mm) with the following eluents: A: H_2O, 0.1% FA + 0.02% TFA and B: MeCN/H_2O (90 : 10, v/v), 0.1% FA + 0.02% TFA, and a linear gradient program: 5% to 100% of B in 15 min, 100% B for 3 min, 100% to 5% B in 0.1 min and 5% B for 6 min, at a flow rate of 0.3 mL/min, at 80 °C.

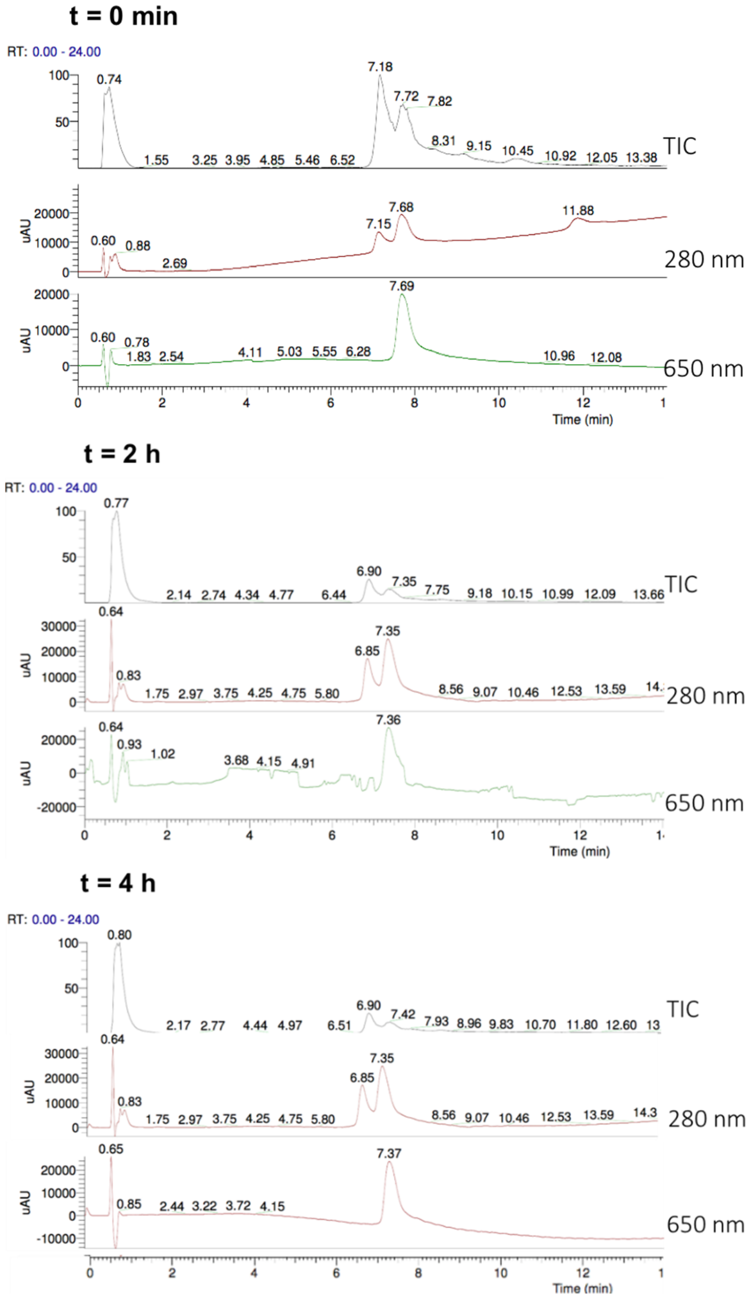

Figure S17. RP-HPLC-MS chromatograms (TIC, 280 nm and 650 nm) of compound DTPA-IRDye700DX-7D12 after 0 min, 2 h and 4 h of incubation in radiolabeling-like conditions.
